# Dataset for the proteomic inventory and quantitative analysis of the breast cancer hypoxic secretome associated with osteotropism

**DOI:** 10.1016/j.dib.2015.09.039

**Published:** 2015-10-23

**Authors:** Thomas R. Cox, Erwin M. Schoof, Alison Gartland, Janine T. Erler, Rune Linding

**Affiliations:** a Biotech Research and Innovation Centre (BRIC), University of Copenhagen (UCPH), Copenhagen DK-2200, Denmark; b Cellular Signal Integration Group (C-SIG), Technical University of Denmark (DTU), Lyngby DK-2800, Denmark; c The Mellanby Centre for Bone Research, The University of Sheffield, Sheffield S10 2RX, UK

**Keywords:** Proteomics, Breast cancer, Bone metastasis, Secretome, Hypoxia, Pre-metastatic Niche, Lysyl Oxidase

## Abstract

The cancer secretome includes all of the macromolecules secreted by cells into their microenvironment. Cancer cell secretomes are significantly different to that of normal cells reflecting the changes that normal cells have undergone during their transition to malignancy. More importantly, cancer secretomes are known to be active mediators of both local and distant host cells and play an important role in the progression and dissemination of cancer. Here we have quantitatively profiled both the composition of breast cancer secretomes associated with osteotropism, and their modulation under normoxic and hypoxic conditions. We detect and quantify 162 secretome proteins across all conditions which show differential hypoxic induction and association with osteotropism. Mass Spectrometry proteomics data have been deposited to the ProteomeXchange Consortium with the dataset identifier PXD000397 and the complete proteomic, bioinformatic and biological analyses are reported in Cox et al. (2015) [1].

**Specifications table**

**Table:** 

Subject area	Biology
More specific subject area	Breast Cancer, Bone Metastasis, Secretome
Type of data	Mass Spectrometry RAW files
How data was acquired	LC-MS/MS on a Q-Exactive Mass Spectrometer (Thermo Fisher Scientific)
Data format	.RAW files
Experimental factors	None applied
Experimental features	The project profiled the expression patterns in hypoxia induced secretomes between MDA-MB-231 parental and MDA-MB-231 Bone Tropic (BT) breast cancer cell lines which have been previously generated by Massague and colleagues (Kang et al. Cancer Cell 2003).
Data source location	Copenhagen, Denmark
Data accessibility	The Mass Spectrometry proteomics data for the article “The hypoxic cancer secretome induces pre-metastatic bone lesions through lysyl oxidase” doi:10.1038/nature14492 [1] have been deposited to the ProteomeXchange Consortium (http://proteomecentral.proteomexchange.org) via the PRIDE partner repository [2] with the dataset identifier PXD000397

***Value of the data***

• This data set will be of value to the scientific community wanting to determine which proteins are secreted from breast cancer cells that metastasize to bone, and those that are regulated by hypoxia.
• The dataset includes quantitative global proteome (Label-free and SILAC) analysis of hypoxic induced secretomes of parental and bone tropic human breast cancer cell lines.
• Differential analysis of secretome changes associated with bone tropism in breast cancer.
• Response of the cancer secretome to hypoxic induction.

## 1 Data, experimental design, materials and methods

### 1.1 Cell lines

The MDA-MB-231 Bone Tropic (BT) 1833 subclone cell line was obtained from J. Massagué at the Memorial Sloan-Kettering Cancer Center. The MDA-MB-231 parental cell line was obtained from the American Type Culture Collection (ATCC) (distributed by LGC Standards). All cell lines were routinely tested for mycoplasma and tested negative for murine pathogens by IMPACT I testing (IDEXX Laboratories).

### 1.2 Collection of hypoxia induced conditioned medium (CM)

For label-free Mass Spectrometry analysis, the MDA-MB-231 parental (wt) cell line and the MDA-MB-231 Bone Tropic (BT) (1833 sub clone derived in Kang et al. [3]) (a kind gift from J. Massagué) were routinely cultured in high glucose Dulbecco׳s Modified Essential Media (DMEM)+GlutaMAX (Gibco #31966-021) with 100 U/ml penicillin and 100 μg/ml streptomycin (Gibco #15140-122), plus 10% dialysed Fetal Bovine Serum (FBS) (Gibco #26400). For conditioned media (CM) collection, cells were grown to 70% confluence, washed with PBS and the media changed to serum-free DMEM and incubated at either 21% oxygen, 5% CO_2,_ 80% humidity (normoxia) or 1% oxygen, 5% CO_2,_ 80% humidity (hypoxia) for 24 h in a Whitley H35 Hypoxystation (Don Whitley Scientific Ltd.). After 24 h, the conditioned media was collected and passed through a 0.22 μM filter to remove floating cells and cellular debris.

For SILAC-based Mass Spectrometry, the MDA-MB-231 parental (wt) and MDA-MB-231 Bone Tropic (BT) cell lines were both grown in light and heavy Stable Isotope Labelling of Amino Acids in Culture (SILAC) DMEM with 100 U/ml penicillin and 100 μg/ml streptomycin, plus 10% dialysed Fetal Bovine Serum (FBS). DMEM (Caisson Labs) lacking arginine and lysine was supplemented with 70 μg/mL light isotope ^12^C-,^14^N-arginine and 140 μg/mL light isotope ^12^C-,^14^N-lysine (Sigma) (R0/K0) or 70 μg/mL heavy isotope ^13^C-,^15^N-arginine and 140 μg/mL heavy isotope ^13^C-,^15^N-lysine (Cambridge Isotope Laboratories) (R10/K8). Cells were grown for 5 passages and label incorporation was assessed through quantification of the number of labelled vs. unlabelled peptides. A minimum of 97–98% labelled arginine and lysine incorporation, with less than 1% proline conversion, was required for subsequent proteomics studies. For conditioned media (CM) collection, cells were grown to 70% confluence, washed with PBS and the media changed to serum-free DMEM and incubated at either 21% oxygen, 5% CO_2,_ 80% humidity (normoxia) or 1% oxygen, 5% CO_2,_ 80% humidity (hypoxia) for 24 h in a Whitley H35 Hypoxystation (Don Whitley Scientific Ltd.). After 24 h, the conditioned media was collected and passed though a 0.22 μM filter to remove floating cells and cellular debris.

### 1.3 Preparation of CMs for Mass Spectrometry

After collection, label-free and SILAC-labelled CMs were reduced in volume using Vivaspin 6 10,000 MWCO, PES Membrane centrifugal concentrators (Sartorius). The remaining protein was fully dissolved in 6 M urea, 2 M thiourea and 10 mM HEPES pH 8, after which exact protein amounts were determined using a Bradford assay. In SILAC-labelled repeats, the two SILAC labels (R10/K8 and R0/K0) were mixed 1:1. In label-free repeats, the samples were left unmixed, but equal amounts of starting material were used for processing. Proteins were reduced in 1 mM DTT (Sigma) for 45 min at room temperature (21 °C), alkylated for 45 min using 5.5 mM chloroacetamide (Sigma), and digested with 1:50 (enzyme:protein ratio) of Mass Spectrometry (MS)-grade trypsin (Sigma) overnight at 37 °C. Peptides were acidified with trifluoroacetic acid at a final concentration of 2%, after which they were desalted on in-house packed C18 StageTips as previously described [4]. Briefly, 2 discs of C18 material (3 M Empore) were packed into a 200 μl pipette tip, and activated with 20 μl of Methanol (HPLC grade) and 20 μl of 80% Acetonitrile, 0.1% FA. The C18 material was equilibrated with 2×20 μl of 1% TFA, 3% Acetonitrile, after which 5 μg of each sample was loaded onto individual StageTips. After washing with 2×20 μl of 0.1% FA, peptides were eluted with 2×40 μl 80% Acetonitrile, 0.1% FA, and concentrated to 5 μl in an Eppendorf Speedvac. This final concentrate was acidified with 4 μl 1% TFA, 2% Acetonitrile for Mass Spectrometry analysis.

### 1.4 Mass Spectrometry acquisition and analysis

5 μg of peptides were loaded onto a 50 cm C18 reverse-phase analytical column (Thermo EasySpray ES803) using 100% Buffer A (0.1% Formic acid in water) at 720 bar, using the Thermo EasyLC 1000 uHPLC system in a single-column setup and the column oven operating at 45 °C. Peptides were eluted over a 4 hour gradient ranging from 6 to 60% of 80% acetonitrile, 0.1% formic acid, and the Q-Exactive (Thermo Fisher Scientific) was run in a DD-MS2 top10 method. Full MS spectra were collected at a resolution of 70,000, with an AGC target of 3×10^6^ or maximum injection time of 20 ms and a scan range of 300–1750 m/z. The MS2 spectra were obtained at a resolution of 17,500, with an AGC target value of 1×10^6^ or maximum injection time of 60 ms, a normalised collision energy of 25 and an underfill ratio of 0.1%. Dynamic exclusion was set to 45 s, and ions with a charge state <2 or unknown were excluded. MS performance was verified for consistency by running complex cell lysate quality control standards, and chromatography was monitored to check for reproducibility.

## 2 Data processing protocol

**Fig. 1:**
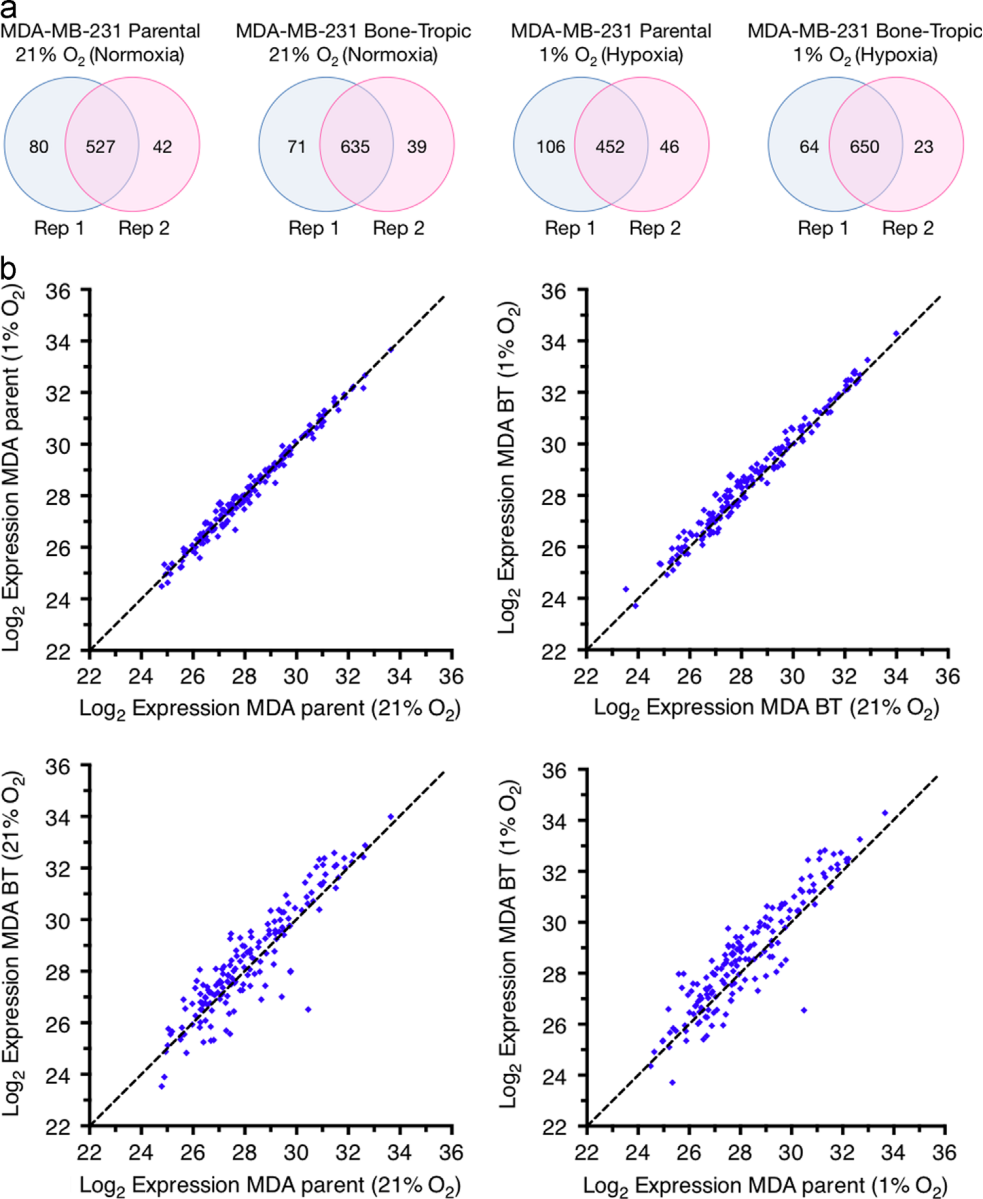
(a) Overlaps between repeats of global secretome analysis in MDA-MB-231 parental and MDA-MB-231 Bone Tropic (BT) cells grown in normoxic (21% O_2_) and hypoxic (1% O_2_) conditions. (b) Log_2_ expression levels under conditions of hypoxia (1% O_2_) and normoxia (21% O_2_) for filtered secreted proteins from the MDA-MB-231 parent and MDA-MB-231 Bone Tropic (BT) 1833 cell line. Data representative of 4 repeats, 2× label-free repeats, and 2× SILAC (standard and reverse-label) repeats. A full list of plotted proteins and their expression levels can be found in Table 1.

Raw data were processed using MaxQuant version 1.5.0.0 [5] (using the human Ensembl GRCh38 database) and Perseus version 1.4. Results were analysed using scripts written in-house in Python and R (deposited in the ProteomeXchange repository alongside data), and tested for statistical significance using the quantile function in the R statistical framework. To ensure high confidence identifications and quantification, a MaxQuant score of >50 and a minimum of two unique peptides per protein seen by tandem MS in all repeats were required. Initial analysis was undertaken using a label-free approach (two repeats) for global pairwise analyses, and data subsequently validated in a standard- and reverse-label SILAC approach (two repeats). Identified intracellular contaminants were removed and secreted proteins retained by using the cellular compartment annotations in Ensembl and PantherDB, and Gene Ontology annotation enrichment for extracellular-associated terms. Overlap between global secretome Madd Spectrometry repeats in are shown in Fig. 1a for each condition. Log_2_ expression data of extracellular secreted proteins in pairwise analysis is shown in Fig. 1b. A full list of detected proteins and their quantitative expression is available in supplementary Table 1. The complete proteomic, bioinformatic and biological analyses are reported in Cox et al. (2015) [1].

## Conflict of interest

The authors declare no conflict of interest.

## Footnotes

Appendix A Supplementary data associated with this article can be found in the online version at 10.1016/j.bios.2014.05.063.
