# Supplementary material for: Dataset for the proteomic inventory and quantitative analysis of the breast cancer hypoxic secretome associated with osteotropism
Source: Data Brief. 2015 Oct 23;5:621–5. doi: 10.1016/j.dib.2015.09.039 (PMC4644255; doi:10.1016/j.dib.2015.09.039)
Supplement: Supplementary file 1 — Supplementary material [file mmc1.docx]

**Table 1 |** Log_2_ expression values from global differential quantitative mass spectrometry based proteomics for each condition.

| **Gene** | **MDA-MB-231**  **Parental**  **(Normoxia)** | **MDA-MB-231**  **Parental (Hypoxia)** | **MDA-MB-231 Bone-Tropic (Normoxia)** | **MDA-MB-231 Bone-Tropic (Hypoxia)** |
| --- | --- | --- | --- | --- |
| ACTA1 | 29.51 | 29.47 | 29.60 | 29.20 |
| ACTN1 | 29.33 | 29.22 | 29.54 | 29.27 |
| ACTN4 | 31.04 | 30.88 | 31.45 | 31.24 |
| AGRN | 28.25 | 28.23 | 29.32 | 29.79 |
| ALDOA | 31.12 | 31.25 | 31.76 | 31.92 |
| ANXA1 | 26.83 | 26.75 | 27.07 | 26.56 |
| ANXA2 | 29.22 | 29.21 | 29.25 | 29.09 |
| ANXA5 | 27.04 | 27.08 | 26.92 | 26.56 |
| APLP2 | 30.39 | 30.35 | 30.88 | 31.28 |
| APP | 30.88 | 30.91 | 30.39 | 30.70 |
| AXL | 29.94 | 30.10 | 30.45 | 31.01 |
| B2M | 31.02 | 31.09 | 32.13 | 32.48 |
| C1QTNF3 | 27.94 | 27.76 | 27.49 | 27.61 |
| CALRx | 26.37 | 26.40 | 26.49 | 26.08 |
| CAPZA1 | 27.12 | 26.87 | 27.11 | 27.21 |
| CCDC80 | 26.08 | 25.78 | 27.35 | 27.98 |
| CD44 | 29.37 | 29.25 | 29.99 | 30.57 |
| CDH13 | 25.74 | 25.88 | 24.83 | 25.35 |
| CDH4 | 26.80 | 26.68 | 25.33 | 25.54 |
| CDK1 | 26.10 | 26.01 | 26.27 | 26.46 |
| CLIC4 | 26.66 | 26.74 | 26.76 | 27.03 |
| CLSTN1 | 29.73 | 29.89 | 29.76 | 30.16 |
| CLTC | 27.98 | 27.48 | 28.15 | 27.91 |
| CNTN1 | 24.89 | 25.34 | 23.90 | 23.71 |
| COL12A1 | 28.83 | 28.94 | 28.69 | 28.86 |
| COL18A1 | 26.39 | 26.15 | 27.47 | 27.59 |
| COL1A1 | 29.15 | 29.25 | 28.77 | 28.65 |
| COL1A2 | 28.21 | 28.18 | 27.43 | 27.74 |
| COL2A1 | 26.68 | 26.70 | 25.85 | 26.27 |
| COL5A1 | 27.36 | 27.65 | 27.83 | 28.36 |
| COL6A1 | 29.43 | 29.54 | 30.29 | 30.55 |
| CPE | 25.19 | 25.37 | 25.66 | 25.84 |
| CSF1 | 26.88 | 27.40 | 28.09 | 28.72 |
| CST3 | 31.07 | 31.30 | 32.38 | 32.83 |
| CTGF | 29.77 | 29.60 | 28.01 | 28.33 |
| CTSB | 27.99 | 28.03 | 28.97 | 29.42 |
| CTSC | 29.76 | 29.78 | 27.97 | 28.52 |
| CTSD | 31.46 | 31.65 | 32.59 | 32.68 |
| CTSL | 28.28 | 28.29 | 28.53 | 28.94 |
| CTSZ | 29.15 | 29.04 | 29.93 | 30.63 |
| CYR61 | 28.52 | 28.03 | 28.94 | 29.07 |
| DAG1 | 28.94 | 29.07 | 29.43 | 29.82 |
| DKK1 | 27.82 | 27.71 | 28.39 | 29.06 |
| ECM1 | 27.57 | 27.86 | 28.01 | 28.29 |
| EDIL3 | 28.38 | 28.57 | 27.90 | 27.78 |
| EHD1 | 26.14 | 26.31 | 27.62 | 27.57 |
| ENO1 | 32.65 | 32.67 | 32.88 | 33.26 |
| EZR | 28.22 | 28.39 | 28.83 | 29.06 |
| FAM3C | 27.82 | 27.83 | 29.29 | 29.42 |
| FKBP1A | 27.69 | 27.96 | 28.59 | 28.97 |
| FLNA | 30.97 | 30.70 | 31.34 | 31.21 |
| FLNC | 28.39 | 28.20 | 28.45 | 28.56 |
| FMOD | 26.69 | 26.88 | 25.31 | 25.94 |
| FN1 | 30.51 | 30.74 | 31.72 | 31.81 |
| G6PD | 26.26 | 25.59 | 28.06 | 27.97 |
| GAPDH | 31.87 | 31.81 | 32.01 | 32.08 |
| GGH | 28.60 | 28.78 | 28.37 | 28.73 |
| GPI | 29.17 | 29.27 | 29.47 | 29.64 |
| HIST1H4A | 27.47 | 27.68 | 27.49 | 27.19 |
| HNRNPK | 27.63 | 26.68 | 27.60 | 27.34 |
| HSP90AA1 | 29.51 | 29.19 | 29.41 | 29.11 |
| HSP90AB1 | 30.64 | 30.23 | 30.72 | 30.45 |
| HSP90B1 | 27.33 | 27.03 | 27.04 | 27.07 |
| HSPA8 | 31.62 | 31.32 | 31.64 | 31.77 |
| HSPG2 | 27.95 | 27.92 | 28.60 | 29.22 |
| HSPH1 | 26.26 | 26.34 | 25.82 | 26.02 |
| HTRA1 | 26.29 | 26.48 | 26.80 | 27.01 |
| IGF2R | 27.91 | 28.00 | 27.27 | 27.40 |
| IGFBP1 | 29.43 | 29.41 | 27.01 | 28.05 |
| IGFBP4 | 30.21 | 30.28 | 30.36 | 30.46 |
| IGFBP7 | 30.77 | 30.64 | 32.05 | 32.45 |
| IL6ST | 25.57 | 25.48 | 25.81 | 25.74 |
| IQGAP1 | 26.96 | 26.42 | 27.17 | 27.11 |
| ITGB1 | 26.72 | 26.69 | 27.15 | 27.16 |
| KAL1 | 25.05 | 25.19 | 25.77 | 26.59 |
| KPNB1 | 27.36 | 27.37 | 27.85 | 27.60 |
| L1CAM | 27.16 | 26.88 | 27.90 | 28.49 |
| LAMA5 | 27.67 | 27.53 | 28.80 | 29.10 |
| LAMB2 | 24.79 | 24.49 | 23.53 | 24.36 |
| LAMC1 | 27.43 | 27.36 | 28.23 | 28.34 |
| LASP1 | 28.09 | 27.87 | 28.74 | 28.94 |
| LDHA | 31.47 | 31.79 | 32.06 | 32.27 |
| LDLR | 26.28 | 26.52 | 26.02 | 26.55 |
| LGALS1 | 29.00 | 28.99 | 30.35 | 30.20 |
| LGALS3BP | 32.15 | 32.15 | 32.24 | 32.48 |
| LMNA | 29.68 | 29.62 | 30.64 | 30.72 |
| LOX | 27.04 | 27.70 | 27.58 | 28.76 |
| LOXL2 | 29.45 | 29.93 | 29.70 | 30.47 |
| LOXL4 | 25.70 | 25.71 | 26.55 | 27.43 |
| MATN2 | 26.63 | 26.67 | 27.17 | 27.83 |
| MET | 27.08 | 27.25 | 27.56 | 28.18 |
| MFGE8 | 26.40 | 26.56 | 25.25 | 25.40 |
| MMP1 | 30.45 | 30.49 | 26.52 | 26.55 |
| MMP14 | 28.11 | 28.33 | 27.85 | 28.54 |
| MSN | 30.92 | 30.86 | 31.37 | 31.48 |
| MYH9 | 29.32 | 29.18 | 30.38 | 30.11 |
| NME2 | 30.58 | 30.42 | 31.05 | 31.21 |
| NPC2 | 26.36 | 26.47 | 26.78 | 26.65 |
| NRP1 | 28.58 | 28.54 | 27.98 | 28.55 |
| NUCB1 | 28.55 | 28.67 | 29.13 | 29.62 |
| P4HB | 27.15 | 26.80 | 27.36 | 27.47 |
| PAM | 25.97 | 26.08 | 26.74 | 27.30 |
| PDIA3 | 27.33 | 27.01 | 27.60 | 27.95 |
| PKM | 32.20 | 32.23 | 32.53 | 32.49 |
| PLAT | 28.23 | 28.75 | 29.54 | 29.83 |
| PLEC | 29.18 | 28.50 | 29.58 | 29.60 |
| PLOD1 | 27.63 | 27.97 | 28.10 | 28.56 |
| PLTP | 26.48 | 26.96 | 26.09 | 26.45 |
| PPIA | 31.84 | 31.93 | 32.39 | 32.73 |
| PPIB | 27.51 | 27.30 | 27.73 | 27.71 |
| PPT1 | 27.28 | 27.33 | 25.69 | 25.96 |
| PRKCSH | 27.26 | 26.91 | 26.78 | 27.28 |
| PSAP | 27.13 | 27.46 | 28.08 | 27.93 |
| PSMA2 | 26.38 | 26.34 | 26.71 | 26.89 |
| PSMA3 | 25.13 | 24.97 | 25.55 | 25.36 |
| PSMA5 | 26.56 | 26.26 | 26.70 | 26.74 |
| PSMA7 | 26.46 | 26.40 | 26.77 | 26.48 |
| PSMD11 | 25.51 | 25.22 | 25.35 | 25.10 |
| PSMD14 | 25.02 | 24.63 | 25.12 | 24.91 |
| PSMD2 | 25.97 | 25.87 | 25.93 | 25.73 |
| PSME1 | 26.59 | 26.65 | 27.27 | 27.39 |
| PSME2 | 26.49 | 26.42 | 26.97 | 26.97 |
| PTPRF | 27.39 | 27.63 | 26.35 | 26.94 |
| PTPRK | 27.19 | 27.45 | 28.19 | 28.52 |
| PTPRS | 25.66 | 25.83 | 25.55 | 25.95 |
| PTX3 | 29.12 | 29.15 | 28.27 | 28.65 |
| PVR | 27.60 | 27.94 | 27.54 | 27.96 |
| PXDN | 28.79 | 29.00 | 27.44 | 27.89 |
| QSOX1 | 31.52 | 31.54 | 31.23 | 31.38 |
| SDC4 | 26.55 | 26.95 | 27.39 | 27.97 |
| SEMA7A | 27.43 | 27.43 | 25.57 | 26.38 |
| SERPINB6 | 24.94 | 24.96 | 24.88 | 25.33 |
| SERPINE1 | 30.88 | 31.12 | 32.34 | 32.75 |
| SERPINE2 | 27.58 | 27.64 | 26.44 | 26.92 |
| SERPINH1 | 25.56 | 25.25 | 25.51 | 25.67 |
| SFN | 27.44 | 27.54 | 29.26 | 29.04 |
| SOD1 | 28.19 | 28.03 | 28.71 | 28.83 |
| SPTAN1 | 26.30 | 26.17 | 26.52 | 26.40 |
| STC1 | 26.47 | 26.92 | 26.99 | 28.02 |
| TCP1 | 26.73 | 26.64 | 26.47 | 26.29 |
| TFRC | 27.82 | 27.66 | 27.26 | 27.55 |
| TGFBI | 27.47 | 27.52 | 29.46 | 29.76 |
| THBS1 | 33.64 | 33.66 | 33.99 | 34.29 |
| TIMP1 | 30.33 | 30.40 | 31.44 | 31.70 |
| TIMP2 | 29.51 | 29.63 | 30.29 | 30.67 |
| TINAGL1 | 26.89 | 27.09 | 27.45 | 28.19 |
| TLN1 | 29.73 | 29.75 | 29.75 | 29.79 |
| TMSB4X | 29.59 | 29.74 | 30.95 | 30.74 |
| TNC | 28.64 | 28.68 | 26.90 | 27.31 |
| TPT1 | 28.19 | 27.72 | 28.07 | 28.08 |
| VASN | 25.63 | 25.95 | 26.90 | 27.15 |
| VCL | 30.43 | 30.45 | 30.62 | 30.77 |
| VCP | 28.79 | 28.54 | 29.27 | 29.14 |
| VIM | 32.58 | 32.17 | 32.43 | 32.36 |
| WDR1 | 28.89 | 28.77 | 29.83 | 29.99 |
| YWHAB | 28.94 | 28.95 | 29.58 | 29.89 |
| YWHAE | 29.69 | 29.60 | 30.04 | 30.02 |
| YWHAG | 27.48 | 27.44 | 28.42 | 28.42 |
| YWHAH | 26.28 | 26.24 | 27.12 | 26.71 |
| YWHAQ | 27.91 | 27.80 | 28.99 | 28.48 |
| YWHAZ | 31.54 | 31.55 | 32.12 | 32.12 |
